# “They were sort of in the room with me”: a qualitative study about callers’ experience of video streaming during medical emergency calls

**DOI:** 10.1186/s13049-024-01317-8

**Published:** 2025-01-08

**Authors:** Siri Idland, Kristin Häikiö, Jo Kramer-Johansen, Magnus Hjortdahl

**Affiliations:** 1 Department of Nursing and Health Promotion, Faculty of Health Sciences, Oslo Metropolitan University, Oslo, Norway; 2 Division of Prehospital Services, Oslo University Hospital, Oslo, Norway; 3 Institute of Clinical Medicine, University of Oslo, Oslo, Norway

**Keywords:** Emergency medical communication center, Dispatcher, Caller, Emergency calls, Medical operator, Video, Video streaming

## Abstract

**Background:**

During the recent years, emergency services in several countries have integrated video streaming into medical emergency calls, and research on the topic has gained increased focus. Video streaming during medical emergency calls may change dispatcher’s perspective of the call and can be a helpful tool for supervising bystanders’ first aid. Little research exists, however, about the caller’s perspective of video streaming during a medical emergency call. With this study, we explore the caller’s experiences with video streaming.

**Methods:**

The study is a qualitative interview study. During a period of five weeks, we recruited respondents from the region of Oslo who had called the medical emergency number 113 and where video streaming had been used by the dispatcher during the call. We conducted 14 semi-structured individual interviews, in-person or digitally on Zoom/Teams, from October to December 2023. The interviews were transcribed verbatim, and we analyzed them drawing on Malterud’s systematic text condensation.

**Results:**

Our material was sorted into three category headings: Increased sense of safety, the unexpected option of video streaming, and emotional discomfort. Most respondents felt comforted knowing that the dispatcher could see and assess the situation visually. Several were also positively surprised that video streaming was an option during the call. Some respondents however felt increased stress during the call due to video streaming. Other respondents reflected on the societal taboo of filming ill or injured persons.

**Conclusion:**

Most respondents experienced video streaming as a positive addition to the medical emergency call and felt comforted knowing that the dispatcher could see the situation. Knowledge of the integration between video streaming and basic communication in a call is nonetheless of great importance, as to not increase stress experienced by the caller. The dispatcher should be sensitive for how the caller will handle video streaming for each call.

**Supplementary Information:**

The online version contains supplementary material available at 10.1186/s13049-024-01317-8.

## Background

For patients with acute disease or injury, medical emergency calls are often a patient’s first meeting with health care. The medical emergency phone number in Norway is 113, and the emergency medical communication center (EMCC) of Oslo is the largest EMCC in Norway, covering approximately 1.5 million of Norway’s 5.5 million inhabitants. The dispatchers answering the calls are either nurses, paramedics, or emergency medical technicians (EMTs) [1]. The traditional way of communicating with the caller has been through regular audio calls. In 2019, an evolution of the communication methods in EMCCs in Norway began: as an additional communication tool between dispatcher and caller during medical emergency calls, video streaming was introduced. It is a live only solution, and the dispatchers themselves choose whether they wish to use video streaming. To activate the solution, the caller must click on a link sent by text message by the dispatcher. The dispatchers are instructed to activate video streaming after they have made the first critical assessment of the situation [2].

Over the last years, there has been an increase in research on video streaming in medical emergency calls. This research includes eventual effects on bystander cardiopulmonary resuscitation (CPR), as well as on resource allocation and bystander first aid. Results imply that video streaming can improve bystander’s technical CPR skills and may improve dispatchers’ recognition rate of the patient’s need for first aid [3–6]. In addition, dispatchers’ experiences with video streaming during the call has been examined in some studies, indicating that video streaming is feasible and, in some situations, can change the dispatcher’s perception of the call [7, 8]. We know less about how callers perceive and experience video streaming during the call. To our knowledge, this study is the first study which explores the caller’s experiences with video streaming during medical emergency calls.

## Methods

We conducted individual semi-structured interviews with 14 respondents who had called the medical emergency number 113, and where video streaming had been used during the call. We chose a qualitative, explorative design because little knowledge on callers’ experiences during medical emergency calls and video streaming exists. This design provides an opportunity to discover new aspects of phenomenon [9]. Semi-structured interviews were chosen mainly due to possible sensitive information in the interviews and that conversation topic might be sensitive for the respondent [10]. We developed an interview guide based on findings from our previous study exploring dispatchers’ experiences with video streaming (Supplementary File 1). After reviewing the first interview, we revised it slightly by adding some new questions, such as: “Can you picture any possible challenges with video streaming?” and “Were you surprised when the dispatcher asked if he/she could activate video streaming?”.

### Recruitment and data collection

Over a period of five weeks, from September 18th until October 23rd, 2023, a text message was sent to all 1336 individuals who had called the medical emergency number 113 in the Oslo region and where video streaming had been used during the call. The text message was sent automatically 24 h after the call had taken place. The receivers of the text messages were first asked whether they were willing to answer some questions regarding quality improvement of 113 calls accessed by a link in the text message. The link led to a short survey in the safe server Nettskjema, which is a web-based survey solution developed by the University of Oslo. In the survey, all respondents filled in demographic data (sex, age and native language), in addition to rating their experience with the 113 call on a scale from 1 to 5 (1—very poor, 5—very good), and their experience with video streaming during the call on a scale from 1 to 5. Age was logged as a continuous variable, and native language had four options: Norwegian, other Scandinavian language, English, Other. For the questions concerning video streaming and the call, we asked specifically how the respondent experienced the conversation, and how the respondent experienced using video streaming during the conversation (see attachment 1 for the survey). At the end of the survey, we presented information about our interview study and invited the respondents to participate in personal interviews regarding the use of video streaming in 113 calls. If they wished to participate, they were asked to leave their first name and phone number.

Among those who wished to participate in the study we applied purposeful sampling as selection strategy of respondents [11]. We used the background data from the survey as a basis for whom to contact and strived to recruit respondents with the widest range possible for all collected background variables, including how they rated their contentment with video streaming. Minors were excluded from interview selection. The interviews were conducted between the end of October and end of December 2023. Each interview lasted from 20 to 40 min and was recorded by Nettskjema Dichtaphone. The respondents chose whether they wanted to be interviewed digitally (Zoom or Teams, by preference) or in-person. Two respondents were interviewed in-person, these interviews were conducted in a meeting room related to the prehospital division at Oslo University Hospital.

### Data analysis

The interviews were conducted, audio recorded and transcribed verbatim by SI. After ten interviews were transcribed, SI and MH read through all material to evaluate the data and to assess whether we thought the study needed additional respondents in order to achieve sufficient information power [12]. We decided that it would be purposeful to collect more data to gain more knowledge on possible negative experiences from video streaming for the caller, and hence conducted four more interviews, after which we felt the information power for our sample was sufficient. For analysis, we drew on systematic text condensation, as developed by Malterud [13]. The approach consists of four steps:

1. Development of initial themes after reading through the material.
2. Sorting data to codes by extraction of meaningful units, development of sub-groups in code groups
3. Condensation of the meaningful units in each sub-group
4. Re-writing of condensations to academic text. Extraction of quotes from the original text for sub-groups. Development of category headings and sub-categories.

Step 1, 2, 3 and 4 was executed mainly by SI, whilst MH participated in step 1. All co-authors participated in step 2 through discussion and comments. Category headings and sub-categories underwent minor changes throughout the process of writing the manuscript. The software NVivo was used during the analytical process for keeping overview of all changes.

### Ethics

Participation was voluntarily. All participants signed a written consent before the interviews took place and received written information about the study and study participants’ rights (Supplementary file 2).

## Results

A total of 245 persons completed and returned the survey. Of these, 49 agreed to be contacted for our study, and 14 were interviewed (for details, see Table 1). All interviews were conducted in Norwegian and translated after transcription.

**Table 1:** Characteristics of interviewed respondents and of respondents of the general quality improvement survey

	Interview respondents, N = 14 (%)		Survey respondents, N = 245 (%)	
*Gender*				
Male	8	(57)	90	(37)
Female	6	(43)	152	(62)
Not defined			3	(1)
*Age group*				
Minors			9	(4)
18–30	4	(29)	36	(15)
31–50	6	(43)	143	(58)
51–70	3	(21)	54	(22)
71 <	1	(7)	3	(1)
*Native language*				
Norwegian	11	(79)	193	(79)
Scandinavian, other			4	(2)
English			4	(2)
Other	3	(21)	44	(18)
*Experience with call*				
Poor	1	(7)	13	(5)
Medium			6	(3)
Good	13	(93)	225	(92)
*Experience with video during call*				
Poor	1	(7)	13	(5)
Medium	2	(14)	8	(3)
Good	11	(79)	224	(92)

We developed three category headings from our qualitative analysis. The categories regard increased sense of safety, the unexpected option of video streaming and discomfort for the caller. See Table 2 for details from the analytical process.

**Table 2:** Overview of initial themes during analytical step 1; groups and subgroups in analytical step 2 during the first and second phase (after discussion with co-authors)

Analytical step 1	Analytical step 2			
Initial themes	Groups (first draft)	Subgroups (first draft)	Category headings (final draft)	Sub-categories (final draft)
Common understanding of the situation	User friendliness	Unexpected technology Possible technological and human barriers	The unexpected option of video streaming	Anticipations to technology Obstacles when activating video streaming
Increased safety	Ease of communication	Difficulties explaining Afraid to say/do the wrong thing What the caller believed was useful to the dispatcher	Increased sense of safety	Feeling safer by showing than telling Shifting the responsibility
User friendliness				
Discomforts	Feeling of safety	Reassurance Guidance		
Guidance	Experienced discomfort	The perspective of the patient Increased stress	Emotional discomforts	The societal taboo of filming ill or injured persons Increased stress in the situation
Experience by the patient of being filmed				

### Increased sense of safety

This category heading emerged into two sub-categories. Findings from the category heading which regarded how video streaming contributed to an increased sense of calm and safety for callers by knowing that the dispatcher could assess the situation visually was named “Feeling safer by showing than telling”. The findings which related to how video streaming led to a feeling of being less alone in the situation for some callers was named “Shifting the responsibility”.

#### Feeling safer by showing than telling

Several of the respondents felt relieved by getting a confirmation from the dispatcher on what they saw, the first aid measures they were doing, and that they knew the dispatcher could see how they executed the dispatcher’s instructions. Several respondents expressed that video streaming made them feel safer and calmer during the call, and that it was easier to explain the situation when they could offer the dispatcher visual information through video streaming in addition to verbal information. One of the respondents described her feeling of increased safety in the following way:

“I just remember that, the feeling of, yes, the feeling that they sort of see me, I’m being seen, that gave me sort of a strength. Ehm, okey, it was just like, yes, I felt it was an extra element of safety. I don’t know how it would have been like with audio only, but they were like, let me see, let me see how it looks.” (Respondent 11, calling about her infant).

One of the respondents said that he had prepared in advance what he was going to tell the dispatcher before he made the call because he worried that he couldn’t explain properly. When the dispatcher then suggested video streaming, the respondent felt relieved and that he could just focus on what he was going to do in the moment. Factors mentioned by several respondents which made explaining the situation more difficult were language problems and nervousness:

“This might sound a bit strange, but maybe especially for me who struggles somewhat with the language, it also helps to show the situation. You sort of feel it is easier to explain what is happening then, they can just see everything for themselves.” (Respondent 4, calling about his girlfriend).

#### Shifting the responsibility

Several of the respondents felt relieved that the dispatcher could observe and evaluate how they executed the dispatcher’s first aid instructions. One of the respondents expressed that she was worried that she had called the EMCC unnecessarily, and that she was misjudging the situation. She felt video streaming contributed positively to the situation, shifting the responsibility for judging the situation to the dispatcher. For her, the situation was too complex to explain verbally.

“But when the video streaming was activated, it was fine, and I thought, now she is assessing this, she is seeing the same as I am, I don’t have to worry, you get scared of not explaining it right (…).” (Respondent 14, calling about a person unknown to her).

Two of the respondents said that talking to the dispatcher through video streaming made them feel like someone was in the room with them, and that they felt less alone in the situation. They felt reassured on an additional level than they would have felt with audio only:

“They were sort of in the room with me, I wasn’t making the decision alone and being the only one giving them feedback, but it was also in a way that they could make a decision when they came into the room.” (Respondent 2, calling about her infant).

### The unexpected option of video streaming

Two sub-categories emerged from this category heading. Findings which concerned how callers received video streaming as a new technology during medical emergency calls was named “Anticipations to technology”, and findings in the category heading which regarded how some callers encountered technical obstacles was named “Obstacles when activating video streaming”.

#### Anticipations to technology

Several of the respondents were surprised that video streaming was an option during the call. They had not heard of the possibility to use video streaming during medical emergency calls and expected a regular call with audio only. Some respondents stated that even though the technology is well known in society, they were surprised that it had been implemented for this purpose. One person said that even though he works with information technology, his mind was set to the fact that audio only was the sole option during the call:

“I was annoyed with myself about how surprised, how positive or surprised I was, on the note that my work is in the same field, so I’m like, why am I so surprised? But I was. You have sort of always known that it’s a phone call, you’re supposed to make a call, that’s what you’ve learned and seen on TV, if you’ve called before that’s what you have experienced.” (Respondent 13, calling about his infant).

In contrast, one of the respondents said that he was surprised it has taken so long for video streaming to be implemented as an option during medical emergency calls, when the technology has been available for many years. He felt it was strange that the solution had not been implemented sooner and found it helpful for both caller and dispatcher. Some believed that persons of higher age might struggle with video streaming during medical emergency calls, while the study’s oldest respondents thought they were coping quite well with the solution. One older respondent stated that she was accustomed to talking to her grandchildren on facetime and that she didn’t think of it as a problem. She felt it was helpful having video when she was calling 113 about her husband:

“I thought it was a good thing, it’s not always easy to explain how my husband is, when they could both hear and see, he was in a lot of pain, they understood that he really was doing bad.” (Respondent 1, calling about her husband).

#### Obstacles when activating video streaming

Two of the respondents emphasized that they wished they had known about video streaming before the call because they felt unprepared to use video streaming. Some of the respondents experienced technical issues. Two respondents experienced that the solution worked at first, but then froze. One of them also experienced issues with the sound. One respondent described his experience of what he felt was a lot of “clicking” to activate video streaming:

“It almost becomes a bit like a salesman trying to sell you something. Follow this link, yes, it’s a lot of clicking. It might be better if the information around it was more common knowledge, being informed about what’s available is important, so that you are prepared to use things like this.” (Respondent 10, calling about his daughter).

### Emotional discomforts

The category heading of emotional discomforts for the caller emerged into two sub-categories. The findings in the category heading which concern how some callers described the notion that filming an ill or injured person often is thought of as a taboo was named “The societal taboo of filming ill or injured persons”. Findings which regarded how other callers experienced increased stress due to video streaming was named “Increased stress in the situation”.

#### The societal taboo of filming ill or injured persons

Two of the respondents reflected on the societal taboo of filming injured or ill persons in public spaces, and how this appeared for other bystanders not knowing that filming was part of the medical emergency call. One of the respondents also believed that this taboo might be an obstacle for doing the filming for certain people. They also thought that situations could occur where both the patient and other bystanders might become aggressive, not understanding the reason for filming. One respondent had negatively associated filming during the call to the issue cases where people film injured or ill persons and posting it on social media platforms. Another of the respondents reflected on how it must have felt for the woman he was helping being filmed:

“It is an uncomfortable situation waiting for the ambulance and then I start to film close up her face, that might have been strange to her.” (Respondent 7, calling about his neighbor).

#### Increased stress in the situation

Two of the respondents felt that both activating the video solution and filming increased stress in the situation they were in. One of the respondents also felt afraid to quit the call when he had to navigate between the call and other windows on his phone to activate the solution. In addition, he said that it also increased stress for his wife, who didn’t understand why he was filming their sick daughter, and that he himself experienced a discomfort by in a way being occupied with filming:

“So, then I received the link, you are a bit shaky and distressed, and I had to start fumbling with that too, right, and then I had to put the camera next to the face of my daughter, and my wife didn’t understand what I was doing with the phone (…) You wish to use your hands to, to hold her, instead of standing there with your phone.” (Respondent 10, calling about his daughter).

One respondent had not wanted to film, but felt she had to, and had a very distressing experience with video streaming. She was terrified and felt that she had called for help but had to film her son being ill instead of receiving help. Her perceptions demonstrates that some people may feel that having to film the situation they are in, increases stress instead of being helpful:

“The problem was that I was a mother, alone, I don’t know what’s happening to my son, I am terrified, and I needed help, professional help, who will come and get him, an ambulance, anything. And instead, I had to film my son.” (Respondent 9, calling about her son).

This respondent reported being afraid and feeling a loss of control; having to film worsened the situation for her.

## Discussion

Most of our respondents experienced video streaming during the medical emergency call as positive, making their explanations of the situation easier. In addition, several respondents felt a greater confidence when the dispatcher could assess the situation visually, which made them feel less alone with decisions. Most also felt that the solution worked well, and many were surprised that video streaming was an option during medical emergency calls. However, some respondents experienced that the technical details didn’t work optimally, which could lead to increased stress. Some also had distressing emotional experiences with video streaming during the call.

In this study, several of the respondents appreciated that the dispatcher could see the situation visually instead of receiving information verbally only from the caller. In a study where dispatchers were interviewed about their experiences with video streaming, some reflected that they thought video streaming contributed to increased reassurance of the caller [8]. Our findings support these reflections. Multiple studies have explored dispatchers’ experiences of assessing a patient or a situation and providing medical advice without any visual inputs, which is often described as challenging [14, 15]. Our study found that the callers in most situations appreciate that the dispatchers are able to see the situation and give advice to the caller based on visual information.

The element of worry about calling the EMCC unnecessarily has been addressed in other studies, where the threshold to call 113 was high, and findings showed that some worried to disturb the EMCC and not exploit public resources unnecessarily [16, 17]. Our study supports these findings, where video streaming was described as a helpful tool in verifying for the caller that calling the medical emergency number was the right thing to do, because the dispatcher could assess the situation visually instead of completely relying on verbal information.

Video streaming is an additional factor that the caller must manage during the medical emergency call. In some cases, video streaming can constitute to additional challenges for the caller, as seen in our findings. Indeed, a dispatcher in our former study reported that she felt video streaming increased stress for the caller: after having activated video streaming, the dispatcher felt that this became too much to handle for the caller [8]. It is worth speculating on whether persisting with the video stream under these conditions generates a risk to the relationship between caller and dispatcher.

In medical emergency calls, establishing the relationship begins when the dispatcher answers the call. As an example, the dispatcher presenting themselves by name is shown to build a relationship, in order to be able to interact with the caller as well as possible [18]. Several studies underline the importance of mindful and empathic communication with the caller [16, 19, 20]. One of these studies showed that how the caller experiences the call did not depend on whether an ambulance was dispatched, but on how they felt they were treated and if they feel heard and seen by the dispatcher [16]. In addition, a training program which was implemented in an EMCC where dispatchers learned techniques to reduce uncertainty and show understanding for the caller in fact reduced time use for calls [21]. Emotional distress makes establishing a relationship of trust even more important than normal situations [22]. If the dispatcher fails to recognize the caller’s emotional state, this can generate conflicts with the caller. This can be unfortunate in several ways, and it can also lead to making the assessment of the patient for the dispatcher more difficult [21].

If the caller experiences highly increased emotional stress due to video streaming, the dispatcher should show understanding and might consider deactivating the video stream. Using video streaming in medical emergency calls still demands high level of communication skills, and we argue that the combination of video streaming and communication techniques should have a focus in the communication training of the dispatchers in the EMCCs. The dispatchers should be sensitive towards the caller’s emotional state before activating video streaming, and not forget the importance of the basic communication strategies and techniques as used in regular calls with audio only. Finally, if the situation of a medical emergency call is in a public place, the reflections from the respondents about how other bystander react to filming not knowing the actual cause for filming are important notions for the dispatchers to be aware of.

### Strengths and limitations

A low number of respondents with a negative experience might be a possible limitation. Of our 14 respondents, only one had a poor experience with video streaming during the medical emergency call. Of the 245 callers completing the survey, 14 had a poor experience with video streaming. We strived to reach additional callers with poor experiences. However, most of the callers who had a poor experience with video streaming did not wish to be contacted for the study. For some of those who had consented and had registered a poor experience, we were not able to establish any contact. The number of persons with a poor experience with video streaming from the survey and of our respondents also align, which is a strength to this study.

It is possible that most of the callers agreeing to be contacted for interviews are positive to video streaming, and that the callers with poor experiences are not interested. We believe that our study is strengthened by having the background data from the survey as a comparison. However, we know that only approximately 20% of the 1336 persons who received the text message after their call to 113 answered the survey. We do not have any knowledge about the callers who did not respond to and cannot be certain that the survey responses mirror the general opinion of the callers in the study’s period.

## Conclusion

The callers describe a variety of positive effects when the dispatcher uses video streaming during medical emergency call. The results show that many experienced the addition of video streaming as an extra element of safety during the call, knowing that the dispatcher could assess the situation visually. However, some respondents also felt video streaming increased stress and had unpleasant experiences with video streaming. Based on our findings, we believe that video streaming can be an important additional tool in medical emergency call but adds to complexity and need for training in communication skills.

## Supplementary Information

Supplementary file 1.

Supplementary file 2.

## Data Availability

No datasets were generated or analysed during the current study.

## Abbreviations

EMCC: Emergency medical communication center
EMT: Emergency medical technician
CPR: Cardiopulmonary resuscitation
