# Supplementary material for: “They were sort of in the room with me”: a qualitative study about callers’ experience of video streaming during medical emergency calls
Source: Scand J Trauma Resusc Emerg Med. 2025 Jan 8;33:5. doi: 10.1186/s13049-024-01317-8 (PMC11715025; doi:10.1186/s13049-024-01317-8)
Supplement: Supplementary file 2 [file 13049_2024_1317_MOESM2_ESM.pdf]

Interview guide

Caller and video streaming project

The interview guide has been prepared on the basis of the results of our previous research project where we interviewed dispatchers at two EMCCs in Norway about their experiences with the use of video streaming.

As there is no research knowledge about the caller's experience, the interview guide will consist of topics with associated open-ended questions.

The interview guide may be revised after each interview, according to the method (semi-structured interview).

We start by asking the caller how long it has been since the incident happened and asking the caller to tell us about the incident.

The following topics will then be addressed:
- Callers' experiences with the use of video – general
- Strengths (positively) with the use of video streaming (communication, relationship and security)
- Challenges (negative) when using video streaming (communication, relationship and security)
- (Experience with the use of video streaming – technical)

Introduce the project. Inform briefly about video in EMCCs. Information about the dictaphone. Inform about confidentiality. They will remain be anonymous in the article. We start by asking the caller how long it has been since the incident happened and asking the caller to tell us about the incident. Information letter and consent.

Research questions	Interview Questions
	Can you tell me briefly about your conversation with 113?
	In the conversation with 113, video streaming was used, how did you experience this?
• Callers' experiences with the use of video streaming – general	Do you think video affected your conversation with 113 in any way?
	What did you think when the person at 113 asked if you could use video?
• Strengths (positively) with the use of video streaming (communication, relationship and security)	Were you stressed by the use of video streaming?
	Did you feel safer or more unsafe after the video streaming was activated? Why do you think you felt this way?

	Do you think anything changed in the conversation after video streaming was activated?<br>(e.g. easier to describe the incident, easier to understand what the person at 113 meant)
• Challenges (negative) when using video streaming (communication, relationship and security)	Do you think there were any challenges with using video streaming?<br>What was difficult, if anything?
	Did you experience any technical challenges with video streaming? E.g. that it was difficult with the link on sms, difficult to hold the phone, or similar

Follow up with "how did you experience this", "what did this do to you", "can you say something more about it" where appropriate. Summarize: Do I understand you correctly in that..., Then I hear that ...,

Change subject: "I would now like to bring up another topic..."

At the end: The purpose of this project is to get your experiences with the use of video streaming as a caller to 113, is there anything else I should ask about or you think we should know?

Debriefing: How did you experience being interviewed about this?
